# Effects of a Dementia Screening Program on Healthcare Utilization in South Korea: A Difference-In-Difference Analysis

**DOI:** 10.3390/ijerph16203837

**Published:** 2019-10-11

**Authors:** Su Jung Lee, Hyun-Ju Seo, Dong Young Lee, So-Hyun Moon

**Affiliations:** 1 College of Nursing, Korea University, Seoul 02841, Korea; supercrystal77@hanmail.net; 2 Department of Nursing, College of Medicine, Chosun University, Gwangju 61452, Korea; shmoon@chosun.ac.kr; 3 Department of Psychiatry, Seoul National University Hospital and Seoul Metropolitan Center for Dementia, Seoul 03080, Korea; selfpsy@snu.ac.kr

**Keywords:** dementia, mass screening, difference in difference, policy analysis

## Abstract

To determine whether Seoul’s dementia screening program increased the rate of diagnosis and the appropriate use of healthcare services for people with dementia, a retrospective data analysis was conducted based on administrative data from the Health Insurance Review and Assessment Service. Two cohorts were constructed to represent the year before Seoul’s dementia screening program began (2007) (control group) and the year after the implementation of the program (2009) (treatment group). A difference-in-difference analysis was used to compare the diagnosis rates, number of clinic visits, and dementia-related drug prescription rates for 4 districts that implemented dementia screening programs between 2007 and 2009 and 14 areas that did not. After the introduction of the program, there was a 55.4% increase in physician-diagnosed dementia. The “average drug cost per patient” increased by 52.2% (Exp(*β*) = 1.522, *p* = 0.0264), the “average outpatient visits per patient” tended to increase by 13.5% (Exp(*β*) = 1.135, *p* = 0.1852), and the “average outpatient treatment fees per visit per patient” tended to increase by 24.4% (Exp(*β*) = 1.244, *p* = 0.0821). The implementation of dementia screening programs led to an increase in healthcare service utilization. Therefore, this program was found to be an effective strategy for reducing undiagnosed dementia cases and encouraging patients to use adequate healthcare services.

## 1. Introduction

Dementia is a condition in which multiple cognitive dysfunctions due to acquired brain diseases cause difficulties in one’s daily or social life [1]. Dementia is usually progressive or chronic in nature and impairs multiple higher cortical functions, including thinking, orientation, memory, comprehension, learning, calculation, judgment, and language capacity [2]. Dementia is the most important condition for an aging society in terms of its cost and impact, and its timely detection is a research, clinical, and political priority [3]. Korea became an aged society in 2018 when its elderly population (people over the age of 65) reached 14.3% of the total population [4]. According to the 2018 Korea Dementia Observatory Report, in 2017, among the elderly population, the number of dementia patients was estimated at 705,473 (male: 254,676, female 450,797), and the prevalence of dementia was 10% [5]. The incidence of dementia increases rapidly with age, and the number of dementia patients is expected to increase dramatically due to the increase in life expectancy [6]. Thus, the number of patients with dementia in Korea is expected to double from 1,000,000 in 2024 to 2,000,000 in 2039, and eventually to more than 3,000,000 in 2050. The number of patients by type of dementia in 2017 was 74.6% with Alzheimer’s disease, 16.7% with other types of dementia, and 8.7% with vascular dementia [5].

According to estimates of the prevalence of dementia using standardized population census data, the distribution of the severity of dementia in 2017 was as follows: 48.4% with very mild dementia, 27.6% mild, 21.9% moderate, and 2.1% severe [7]. The proportions of very mild and mild dementia accounted for the majority, suggesting that early screening for dementia and appropriate medical care are necessary.

Early detection of dementia is important for optimal disease management [8]. Though the number of dementia patients worldwide is increasing rapidly, approximately three-quarters of the world’s dementia population has not yet been diagnosed and the majority of dementia sufferers are diagnosed in the later stages of the disease; this results in a significant treatment gap [9]. A systematic review also showed that the true prevalence of delayed and missed diagnoses of dementia appears to be high, and this leads to a loss of treatment opportunities and an increased burden on patients and caregivers [10].

Early diagnosis of dementia is crucial because only through receiving a diagnosis can patients access available nondrug and drug therapies that may enhance their cognition and improve their quality of life [9]. For example, medications for Alzheimer’s disease (anticholinesterases and memantine) have been shown to improve cognitive dysfunction and activities of daily living (ADL) function temporarily and decrease the development of behavioral problems [11]. In addition, a timely diagnosis of dementia is also important for effective management because patients and their families can receive support, plan for the future, and be motivated to live a healthier life [12,13,14,15,16]. Thus, medical practitioners working with older people (especially people over 75 years old) should be careful about cognitive decline and recommend early dementia identification of the symptoms of cognitive decline; meanwhile, screening of the general population is not recommended [17].

The Dementia Support Centers (DSCs) in Seoul, Korea have been working to prevent and screen dementia and provide appropriate medical and social services to patients with dementia by implementing and supporting a dementia management project in 25 districts in Seoul, starting with 4 districts in 2007. The detection of dementia patients is performed in three stages. Step 1 is a dementia screening test using the Korean version of the Mini Mental State Examination for Dementia Screening at public health centers and dementia centers; step 2 is a detailed diagnostic test, such as neurocognitive testing and assessment by medical specialists at dementia centers; and step 3 is a confirmed test by blood analysis or neuroimaging at a hospital. According to previous studies, from 2008 to 2012, the incidence of dementia in Seoul was 3.3% [18]. In addition, 697,968 elderly people in Seoul were screened, and 31,501 were diagnosed as having dementia, reporting an average annual detection rate of 4.35% during 2008–2012 [19].

The purpose of this study was to evaluate the effect of the dementia screening program (DSP) conducted by the DSCs on healthcare service utilization. While there are debates about the pros and cons of screening for dementia, such as the risk of misdiagnosis, several studies on cost effectiveness have recently attempted to evaluate the outcomes of dementia screenings [20,21,22,23]. However, the effects of DSP analysis are based on a wide range of assumptions, and additional evidence on these healthcare service utilization topics is required. Therefore, this study aimed to evaluate the effect of the DSP on healthcare service utilization for people 60 years or older residing in the districts where the DSP was introduced in 2008 by comparing it to another population group of people 60 years or older living in districts that did not introduce the DSP.

Specifically, we compared Seoul’s autonomous regions regarding the changes in and effects on the volume and strength of healthcare service utilization from the data on registered subjects in 4 districts where the DSP was introduced and 14 districts where it was not.

## 2. Methods

### 2.1. Study Design

This study used registry data from the Seoul Metropolitan Center for Dementia (SMCD, Jongno-gu, South Korea) and Electronic Data Interchange (EDI) request data from the Health Insurance Review and Assessment Service (HIRA) between 1 January 2007 and 31 December 2009. South Korea’s health insurance system is a national unified system that covers all citizens. It includes mandatory medical care and health insurance supplied by healthcare providers. The HIRA reviews medical fee claims about health insurance and assesses the adequacy of healthcare services. Claims data are collected when the healthcare provider submits a claim to the HIRA to receive reimbursement for services provided to the patients. After reviewing the claim, the HIRA submits the result to the health institution and the National Health Insurance Service for payment. For claims data management, the HIRA’s EDI database contains management and operation codes [24,25]. The registry data were linked to EDI request data and described the records of medical services associated with dementia diagnoses for the sample groups over two years. The difference-in-difference (DID) method was used to estimate the effects of the DSP on the volume and strength of healthcare service utilization for those aged 60 years or over before and after the introduction of the DSP in Seoul. 

**Figure 1:**
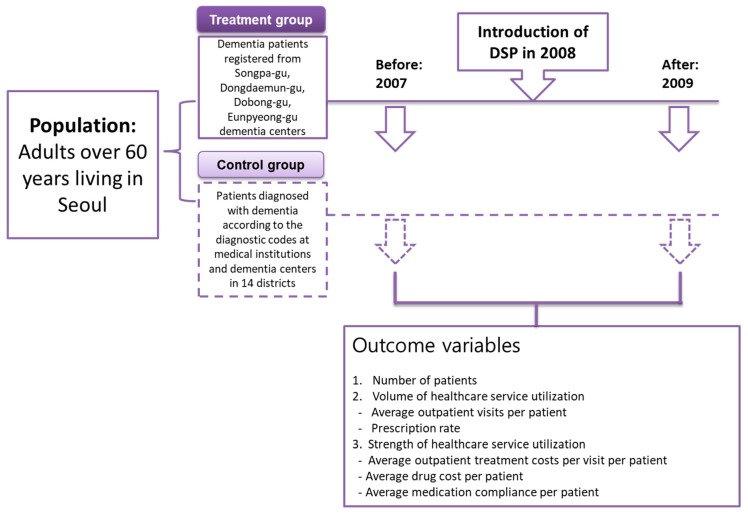
Overview of study design to evaluate the effects of the introduction of a dementia screening program (DSP).

The treatment group in this study consisted of people aged 60 years or older who lived in 4 districts (Songpa-gu, Dongdaemun-gu, Dobong-gu, and Eunpyeong-gu) provided with a DSP, while the control group consisted of those aged 60 years or older who lived in the 14 other districts (Gwangjin-gu, Gurogu, Gangbukgu, Geumcheongu, Junggu, Gangseo-gu, Yeonggu, Yongsan-gu, Jongno-gu, Jungnanggu, Yeongdeungpo, Nowongu, Seodaemun-gu, and Gangnam-gu), where a DSP was not available between 2007 and 2009. The participants were those who had experienced outpatient treatment for dementia within the seventh rank of a major or minor diagnosis in the three years from 1 January 2007 to 31 December 2009. In South Korea, all patient data are submitted for health insurance claims by healthcare providers to the HIRA, and the HIRA’s claim data are national data compiled from across the country. Participants in this study used the HIRA’s patient data that included diagnosis of dementia in the claims submitted by the healthcare provider. The research data included 2007 and 2009 cohorts. The 2007 cohort represented the year before the introduction of the DSP and the 2009 cohort represented the year after the introduction of the DSP (Figure 1). 

In detail, we compared healthcare service utilization before (2007) and after (2009) the dementia project by dementia management subjects diagnosed through the 2008 dementia screening and management project. Then, dementia patients in the treatment group were compared with those in the control group, who were charged for outpatient reimbursement using dementia diagnosis codes by healthcare institutions located in the 14 districts. The EDI data came from the consigned hospitals, the cooperative hospitals of each district’s community health center, and the medical institution where each district is located.

### 2.2. Outcome Variables

In this study, we investigated the extent to which a new DSP for people aged 60 or over residing in a community resulted in changes in healthcare utilization regarding outpatient utilization volume and strength [26]. Our hypothesis assumed that the number of patients diagnosed with dementia through DSP would increase, resulting in increased outpatient utilization volume and strength, such as the prescription of dementia medication and hospital visits. The utilization volume included the number of patients diagnosed with The International Statistical Classification of Diseases and Related Health Problems -10 codes F00, G30, F01, F02, and F03. Because this diagnosis code is a dementia-specific code, even if the patient has other diseases, the purpose of the healthcare service utilization can be assumed to be for dementia care. In addition, we investigated average outpatient visits per patient diagnosed with the same codes, and the number of patients prescribed at least one of the following four drugs: donepezil HCL, galantamine hydrobromide (as galantamine), memantine HCL (as memantine), and rivastigmine. In terms of the strength of healthcare service utilization, we measured average outpatient treatment costs per visit per patient, defined as the total cost of outpatient treatment divided by the number of patients and number of visits; the average drug cost per patient, calculated as the total drug cost divided by the number of patients; and the average medication compliance per patient, defined as cumulative medication adherence (CMA, calculated as the total number of days of medication dispensed divided by the total number of days between the first and last dementia prescriptions in the observation period). 

### 2.3. Statistical Analysis

This research used the DID method of analysis to identify the impact of a DSP on healthcare service utilization in a general population aged 60 years or older. The change in healthcare utilization in the treatment group before and after the introduction of the DSP, minus the corresponding change in the control group, provided an estimate of the impact of the DSP on healthcare use. The assumption of the DID is as follows. Most of the treatment groups should be affected by the policy interventions, the control group should be largely unaffected, and factors other than the effects of the introduction of the program should be similar between the treatment and control groups. This study examined two time periods for each individual and utilized an unobserved effects cohort data model. The observed outcome equation in terms of the group and time period indicators to obtain the standard DID estimating equation [27] was as follows:

T: Treatment group, C: Control group, $t_{0}$: pre-DSP (2007), $t_{1}$: post-DSP (2009)
$Y_{0}^{T},{\;Y}_{1}^{T}$: Healthcare utilization in the treatment group; $Y_{0}^{c},{\;Y}_{1}^{c}$: Healthcare utilization in the control group
  $$Y_{i}=\;\alpha +\beta T_{i}\;+\;\theta t_{i}\;+\;\delta \left(T_{i}\times t_{i}\right)\;+\;\varepsilon_{i}$$
α: intercept, β: difference between treatment group and control group, θ: the time trend, *i:* person
$T_{i}$: the time-invariant difference in outcomes between the two groups
$t_{i}$: the combined effects of any unmeasured covariates that changed between the two periods but affected the outcomes the same way in both groups
δ: true effect of treatment under the common trend assumption, $\varepsilon_{i}$ = random error
  $$\delta_{DID}\;=E(Y_{1}^{T})-E\left(Y_{0}^{T}\right)-\left[E\left(Y_{1}^{c}\right)-E(Y_{0}^{c}\right)]$$

A DID model simulates a random assignment experiment with treatment and comparison groups. Program effects were estimated by comparing changes in healthcare utilization categorized by volume and strength between the pre- (2007) and post-DSP (2009) years for eligible and ineligible members, then adjusting for covariates and secular trends in healthcare utilization. Individual-level random effects were included to prevent bias from unobserved differences in motivation and preferences that influence participation and, ultimately, program outcomes. We controlled for age; sex; major diagnosis of dementia; comorbidity, such as the presence of hypertension; diabetes; depression; stroke; type of institution; and clinical department in the model. Information on patient demographics and institutional characteristics in the treatment group was obtained from patients’ registry files from the SMCD, whereas control group information was derived from the EDI request data of the HIRA. For the number of outpatient visits and reimbursement costs for outpatient services, prescribed drug costs were log-transformed to assume a normal distribution. Statistical analyses were performed using SAS ver. 9.13 (SAS Institute, Carry, NC, USA) and the statistical significance was set at *p* < 0.05.

### 2.4. Ethics

Informed consent was obtained from individuals in the treatment group of the community health center. Then, the consent forms were collected in the SMCD database. For the control group, the acquisition of EDI data was approved by the HIRA, and the EDI request data were anonymized; therefore, the need for patient consent forms was waived. This study was approved by the Chosun University Review Board on 1 May 2013 (IRB No. IRB-13-014).

## 3. Results

In order to analyze the policy effects of the DSP, 26,531 cases of HIRA claims and registry files from the SMCD from 2007, before the implementation of the dementia project, and 42,920 cases of HIRA claims and registry files from the SMCD from 2009, after the dementia project, were compared. A DID analysis was used to compare the autonomous districts where the dementia project was implemented and those where it was not. 

We created a cohort of 13,981 dementia patients enrolled in 2007, which represents the year before the introduction of the DSP. Of these, 107 were in a treatment group in one of the four districts that provided the DSP in 2008. The mean age of the 2007 cohort of dementia patients was 75.15 years (standard deviation (SD): 7.65), 66.74% had a major diagnosis of Alzheimer’s disease, and 67.21% were female (Table 1a). 

We created another cohort of 25,371 dementia patients registered in 2009. Of these, 253 were in a treatment group in one of the four districts that received the DSP in 2008. The control group consisted of 25,119 patients from the other 14 districts. The mean age of the 2009 cohort was 75.56 years (SD: 7.72), 62.63% had a major diagnosis of Alzheimer’s disease, and 67.35% were female (Table 1b).

### 3.1. Differences in Healthcare Service Utilization before and after the Introduction of the DSP in the Treatment and Control Groups

In the analysis of the effects of the dementia management program using the DID method for subject areas of the dementia project and nonsubject areas, the ratio of the difference between the two groups in the “number of patients who were diagnosed with dementia” was 55.4%, which was higher in the subject areas than in the nonsubject areas (Table 2).

**Figure 2:**
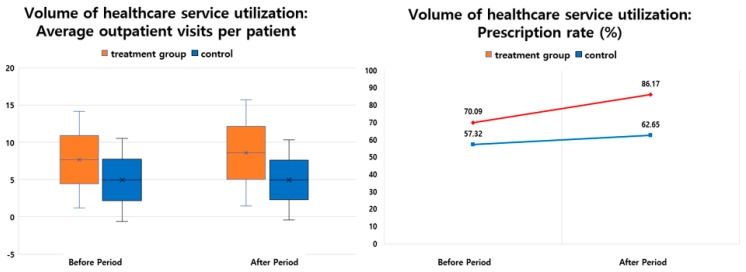
Outcomes of interest regarding volume of health service utilization.

Regarding the estimate indicator of the “volume of healthcare service utilization”, the ratio of the DID between the two groups for “average outpatient visits per patient” was 11.42%, which was higher in the subject areas than in the nonsubject areas. The “prescription rate” was 92.78% higher in the subject areas than in the nonsubject areas (Table 2, Figure 2).

**Figure 3:**
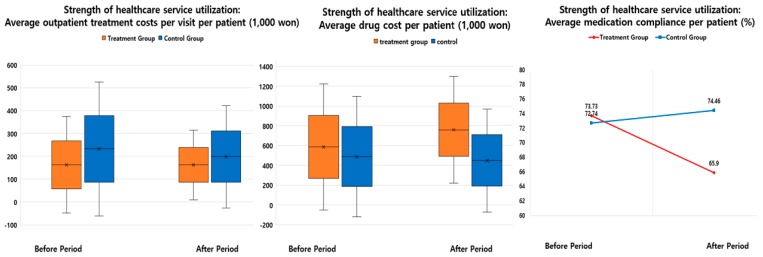
Outcomes of interest regarding strength of healthcare service utilization.

Additionally, regarding the estimate indicator of “strength of healthcare service utilization”, the ratios of the DID between the two groups for “average outpatient treatment costs per visit per patient” and “average drug cost per patient” were also consistently higher in the subject areas than in the nonsubject areas. However, the ratio of the DID between the two groups for “average medication compliance per patient” was −12.97%, which was lower in the subject areas (Table 2, Figure 3).

### 3.2. Effects of the Introduction of the DSP on Healthcare Utilization

A regression analysis using the DID method was performed to obtain the regression coefficients for the interaction between groups before and after the implementation of the DSP by adjusting other explanatory variables (age, sex, major diagnosis, comorbidities, type of institution, and clinical department). In the case of “average outpatient visits per patient”, “average outpatient treatment fees per visit per patient”, and “average drug cost per patient”, the data were shifted to the right and analyzed after being log-transformed to take the normality into account.

The results of the multiple linear regression analysis on the “implementation of DSP × before/after the introduction of the DSP”, showing the effect of the introduction of the project itself, revealed an increase of 52.2% in the “average drug cost per patient”, and this was statistically significant (Exp(β) = 1.522, *p* = 0.0264) (Table 3). Other outcome measures also showed an increase in healthcare utilization (“average outpatient visits per patient”: 13.5% and “average outpatient treatment fees per visit per patient”: 24.4%), but they were not statistically significant (Table 3).

## 4. Discussion

To the best of our knowledge, this study was the first to evaluate the effects of the introduction of a dementia management policy based on administrative data in South Korea. To date, relatively few studies have focused on the effects of early detection on healthcare services [8]. This study was a retrospective analysis based on data from a comprehensive patient registry from 2007 to 2009 from the SMCD as well as EDI request data from the HIRA for the purpose of analyzing the effect of a dementia management program in Seoul on healthcare utilization. The claim data are the dementia-specific code data for which medical fees were charged for dementia care. Therefore, we could assume that the purpose of the healthcare service utilization was for dementia care, even if they had other comorbidities. In particular, by conducting a regression analysis using the DID method, we examined the healthcare utilization outcome variables of the treatment and control groups from 2007 to 2009 and found significant changes due to the positive effects of the DSP in Seoul. 

As a result of the comparison, it was found that the number of patients diagnosed with dementia increased by 55.4% in the subject areas compared with the nonsubject areas. The rate of diagnosis of dementia in Korea increased to 73.6% in 2015 from 51.3% in 2010, but the rate of dementia patients not yet diagnosed reached 26% in 2015 [7,28]; thus, this study supports the notion that the diagnosis rate of dementia can be improved if the DSP is extended. 

Of note is that the logistic regression analysis using the DID model showed that the “average drug cost per patient” increased by 52.2% (Exp(β) = 1.522, *p* = 0.0264), the “average outpatient visits per patient” by 13.5% (Exp(β) = 1.135, *p* = 0.1852), and the “average outpatient treatment fees per visit per patient” by 24.4% (Exp(β) = 1.244, *p* = 0.0821). These outcome variables represent the volume and strength of healthcare service utilization, and these results are evidence that the implementation of the DSP not only enhanced the diagnosis of dementia patients but also increased healthcare utilization related to diagnosed dementia. In the comparison of the prevalence rate of dementia in Korea in 2008 and 2012, the prevalence of very mild and mild dementia was 68% and 58.8%, respectively, and this rate was higher than the prevalence of moderate and severe dementia [7]. Therefore, for the early detection and management of dementia, the DSP needs to be continuously implemented. 

The Korean government declared a “war on dementia” in 2008 and has prepared a national dementia plan every five years [23]. As a means of restricting disease disability, mass DSPs can lead to early diagnosis, proper treatment, and, potentially, disability limitation [8]. Therefore, this quasi-experimental evaluation of such a DSP can significantly contribute to the research and practice related to dementia.

The limitations of this study are as follows. First, the comparison of healthcare utilization between the target and nontarget areas of the DSP in 2008 was based on the cost data of claims for medical care charged (requested) by healthcare institutions belonging to each autonomous region. Due to the nature of the health insurance claim data, it was possible to obtain the location of the medical institutions in each district, but it was not possible to acquire resident registration number data for the over-60 population group living in each area. Since the medical institutions of the local districts include general hospitals, such as the Big Five hospitals, we cannot exclude the possibility that subjects not residing in the relevant autonomous regions among the healthcare utilization subjects were included. Second, medication compliance among the outcome variables was assumed to be the steady taking of drugs for Alzheimer’s. That is, the total number of prescription days for at least one of the four drugs for Alzheimer’s disease was taken as a molecule. If a drug was prescribed in the second half of the year, in the CMA calculation of medication compliance, the denominator would become smaller and, therefore, the CMA value may have been calculated to be high, which may have resulted in an overestimation of overall medication compliance. Lastly, this study evaluated the related medical benefits by analyzing the increase in the number of dementia patients and the healthcare utilization outcome due to DSP, but it did not identify the patients’ psychological benefits (e.g., quality of life) or harms (e.g., anxiety). This program is aimed at reducing the diagnosis gap, which is the difference between the estimated number of dementia patients and the actual number of patients who are receiving healthcare services, so that undiagnosed dementia patients receive proper care. Studies have reported that there are multiple benefits, such as reducing the disease burden by allowing early diagnosis, treatment, and appropriate interventions [9,11,29,30]. The main issue in the debate about the harms of dementia diagnosis is mainly the risk of misdiagnosis, buy the DSP program in this study is comparatively accurate because it is diagnosed in three stages (screening test, detailed diagnostic test, and confirmation test). In addition, the dementia support center in the treatment group of four districts provided support programs, such as family education and cognitive health programs, with DSPs.

Despite these limitations, this study has the advantage of being a comprehensive data survey of Seoul that can be representative of the medical use effect of the Korean dementia screening test. Notably, this is the first study to evaluate the effects of the introduction of the DSP derived from the dementia management policy based on administrative data on healthcare utilization. In addition, this study employed a quasi-experimental design using a DID model to estimate the policy effect and show whether the policy goals were achieved in a methodologically robust way. This study provides strong scientific evidence for the further development of a program for screening dementia in Korea. Therefore, if studies can be carried out continuously every year, it will be possible to calculate an index of medical use monitoring results, such as the rate of prescriptions and the drug costs for dementia.

This study is expected to be used as a basis for decision-making related to an evidence-based health policy for the implementation of a national dementia management plan. The conception of such a plan will need to involve an examination of the status of healthcare service utilization related to dementia using health insurance data obtained through the Seoul dementia management project and data on the current status of domestic dementia treatment. 

Future studies on healthcare services, including cost-effectiveness studies and endpoint outcome studies on delayed deterioration in disease progression, reductions in the institutional admission rate, mortality due to dementia, and improvements in quality of life for both dementia patients and caregivers, should be conducted proactively and systematically. Among the factors contributing to the underdiagnosis issue, taking into account delays in seeking a primary care physician due to the fear of stigma [8], information campaigns for the DSP that can address such perceptions are also important. Finally, there is also a need for research on strategies to increase medication compliance that take into account the quality of care. 

## 5. Conclusions

The implementation of DSPs led to improvements in healthcare service utilization, such as the diagnosis rate of dementia, medication prescriptions for dementia, and reimbursement costs for outpatients. Therefore, this program was an effective intervention for reducing the increasing burden of dementia in an aged society.

## Figures and Tables

**Table 1:** (**a**) Participant characteristics at program introduction (2007). (**b**) Participant characteristics by program introduction (2009).

**Variables **	**Category**	**(a)**						***p*** **-Value**
		**Treatment Group**		**Control Group**		**All Patients**		
		**(N = 107)**		**(N = 13,874)**		**(N = 13,981)**		
		**Mean (SD) or n (%)**	**Range**	**Mean (SD) or n (%)**	**Range**	**Mean (SD) or n (%)**	**Range**	
Age (years)		75.22 (7.56)	60–95	75.15 (7.65)	60–109	75.15 (7.65)	60–109	0.9154 (t)
Age group (years)	60–64	9 (8.41)		1215 (8.76)		1224 (8.75)		0.9669 (c)
	65–69	15 (14.02)		2261 (16.30)		2276 (16.28)		
	70–74	25 (23.36)		3074 (22.16)		3099 (22.17)		
	75–79	24 (22.43)		3181 (22.93)		3205 (22.92)		
	≥80	34 (31.78)		4143 (29.86)		4177 (29.88)		
Sex	Male	32 (29.91)		4552 (32.81)		4584 (32.79)		0.5240 (c)
	Female	75 (70.09)		9322 (67.19)		9397 (67.21)		
Major diagnosis	Alzheimer’s disease	83 (77.57)		9248 (66.66)		9331 (66.74)		0.0208 (c)
	Vascular dementia	14 (13.08)		1889 (13.62)		1903 (13.61)		
	Other dementia	10 (9.35)		2737(19.73)		2747(19.65)		
Comorbidities	Hypertension	16 (14.95)		1305 (9.41)		1321 (9.45)		NE
	Diabetes	4 (3.74)		476 (3.43)		480 (3.43)		
	Depression	16 (14.95)		2270 (16.36)		2286 (16.35)		
	Stroke	20 (18.69)		1624 (11.71)		1644 (11.76)		
Type of institution	General hospital	52 (48.60)		10,170 (73.30)		10,222 (73.11)		<0.0001 (c)
	Hospital	28 (26.17)		777 (5.60)		805 (5.76)		
	Clinic	27 (25.23)		2818 (20.31)		2845 (20.35)		
	Public health facility	0 (0.00)		109 (0.79)		109 (0.78)		
Clinical department	Neurology	56 (52.34)		5729 (41.29)		5785 (41.38)		0.0718 (c)
	Psychiatry	37 (34.58)		5187 (37.39)		5224 (37.36)		
	Internal medicine	5 (4.67)		958 (6.91)		963 (6.89)		
	General medicine	6 (5.61)		762 (5.49)		768 (5.49)		
	Others	3 (2.80)		1238 (8.92)		1241 (8.88)		
**Variables **	**Category**	**(b)**						***p*** **-Value**
		**Treatment Group**		**Control Group**		**All Patients**		
		**(N = 253)**		**(N = 25,119)**		**(N = 25,372)**		
		**Mean (SD) or n (%)**	**Range**	**Mean (SD) or n (%)**	**Range**	**Mean (SD) or n (%)**	**Range**	
Age (years)	Mean (SD)	76.49 (7.45)	60–97	75.55 (7.72)	60–110	75.56 (7.72)	60–110	0.0557 (t)
Age group (years)	60–64	13 (5.14)		1996 (7.95)		2009 (7.92)		0.2484 (c)
	65–69	36 (14.23)		3930 (15.65)		3966 (15.63)		
	70–74	50 (19.76)		5533 (22.03)		5583 (22.00)		
	75–79	63 (24.90)		5722 (22.78)		5785 (22.80)		
	≥80	91 (35.97)		7938 (31.60)		8029 (31.65)		
Sex	Male	73 (28.85)		8210 (32.68)		8283 (32.65)		0.1960 (c)
	Female	180 (71.15)		16,909 (67.32)		17,089 (67.35)		
Major diagnosis	Alzheimer’s disease	214 (84.58)		15,676 (62.41)		15,890 (62.63)		<0.0001 (c)
	Vascular dementia	22 (8.70)		4271 (17.00)		4293 (16.92)		
	Other dementia	(0.00)		5172 (20.59)		5172 (20.38)		
Comorbidities	Hypertension	27 (10.67)		2563 (10.20)		2590 (10.21)		NE
	Diabetes	9 (3.56)		863 (3.44)		872 (3.44)		
	Depression	52 (20.55)		3190 (12.70)		3242 (12.78)		
	Stroke	42 (16.60)		3719 (14.81)		3761 (14.82)		
Type of institution	General hospital	110 (43.48)		18,910 (75.28)		19,020 (74.96)		<0.0001 (c)
	Hospital	108 (42.69)		1121 (4.46)		1229 (4.84)		
	Clinic	32 (12.65)		4787 (19.06)		4819 (18.99)		
	Public health facility	3 (1.19)		301 (1.20)		304 (1.20)		
Clinical department	Neurology	155 (61.26)		12,437 (49.51)		12,592 (49.63)		<0.0001 (c)
	Psychiatry	73 (28.85)		7289 (29.02)		7362 (29.02)		
	Internal medicine	8 (3.16)		1778 (7.08)		1786 (7.04)		
	General medicine	11 (4.35)		1061 (4.22)		1072 (4.23)		
	Others	6 (2.37)		2554 (10.17)		2560 (10.09)		

SD = Standard deviation. Percentages (%) are based on the total number of patients in each group (N). *p*-value: Chi-squared test (c), Fisher’s exact test (f), or two-sample *t*-test (t). Subjects experienced multiple events under the comorbidities category.

**Table 2:** Healthcare service utilization before and after the introduction of the dementia screening program (difference-in-difference analysis).

	Treatment Group				Control Group				DID	DIR
	Before Period	After Period	Difference	Ratio	Before Period	After Period	Difference	Ratio		
	A	B	E = B − A	G	C	D	F = D − C	H	E − F	G − H
Number of patients	107	253	146.0	136.45	13,874	25,119	11,245	81.05	−11,099	55.40
Volume of healthcare service utilization										
Average outpatient visits per patient	7.67 ± 6.48	8.55 ± 7.13	0.87	11.37	4.95 ± 5.56	4.95 ± 5.35	0.00	−0.05	0.88	11.42
Prescription rate (%)	75 (70.09)	218 (86.17)	143.0	190.67	7953 (57.32)	15,738 (62.65)	7785	97.89	−7642	92.78
Strength of healthcare service utilization										
Average outpatient treatment costs per visit per patient (₩1000)	162.61 ± 211.27	162.40 ± 151.82	−0.21	−0.13	232.39 ± 292.73	197.91 ± 224.70	−34.48	−14.84	34.27	14.71
Average drug cost per patient (₩1000)	586.02 ± 637.79	760.06 ± 539.64	174.04	29.70	488.43 ± 607.81	449.64 ± 520.37	−38.79	−7.94	212.83	37.64
Average medication compliance per patient (%)*	73.73	65.90	−7.83	−10.62	72.74	74.46	1.71	2.35	−9.54	−12.97

G = E/A × 100. H = F/C × 100. DID = Difference-in-difference. DIR = Difference-in-ratio. Increase rate compared to 2007: G = (B − A/A) × 100, H = (D − C/C) × 100. *Medication compliance was calculated as cumulative medication adherence (CMA).

**Table 3:** Effects of the introduction of the DSP on the healthcare utilization outcome variables.

Variables		β	Exp(β)	SE	95% Confidence Interval		*p*-Value
					Lower	Upper	
Average outpatient visits per patient	Groups						
	Control group		1				
	Treatment group	0.355	1.426	0.080	0.198	0.513	<0.0001
	Period						
	2007		1				
	2009	0.064	1.066	0.009	0.047	0.081	<0.0001
	Groups × Period	0.127	1.135	0.096	−0.061	0.315	0.1852
Prescription rate (%)	Groups						
	Control group		1				
	Treatment group	−0.406	0.206	0.666	0.4448	0.9974	<0.0001
	Period						
	2007		1				
	2009	−0.171	0.023	0.843	0.8062	0.882	0.0485
	Groups × Period	−0.144	0.248	0.866	0.5329	1.4061	0.5599
Average outpatient treatment fees per visit per patient (₩1000)	Groups						
	Control group		1				
	Treatment group	−0.154	0.857	0.105	−0.360	0.052	0.1435
	Period						
	2007		1				
	2009	−0.086	0.918	0.012	−0.108	−0.063	<0.0001
	Groups × Period	0.218	1.244	0.126	−0.028	0.464	0.0821
Average drug cost per patient (₩1000)	Groups						
	Control group		1				
	Treatment group	0.153	1.165	0.163	−0.166	0.472	0.3470
	Period						
	2007		1				
	2009	−0.073	0.929	0.019	−0.110	−0.037	<0.0001
	Groups × Period	0.420	1.522	0.189	0.049	0.791	0.0264
Average medication compliance per patient (%) **	Groups						
	Control group		1				
	Treatment group	0.052	1.054	0.089	−0.122	0.227	0.5578
	Period						
	2007		1				
	2009	0.031	1.032	0.014	0.005	0.058	0.0203
	Groups × Period	−0.170	0.844	0.130	−0.425	0.085	0.1915

** Medication compliance was calculated as cumulative medication adherence (CMA).
